# Molecular Epidemiology of Multidrug-Resistant Tuberculosis, New York City, 1995–1997

**DOI:** 10.3201/eid0811.020288

**Published:** 2002-11

**Authors:** Sonal S. Munsiff, Trina Bassoff, Beth Nivin, Jiehui Li, Anu Sharma, Pablo Bifani, Barun Mathema, Jeffrey Driscoll, Barry N. Kreiswirth

**Affiliations:** * New York City Department of Health, New York, New York, USA; † Centers for Disease Control and Prevention, Atlanta, Georgia, USA; ‡ Public Health Research Institute TB Center, Newark, New Jersey, USA; § Wadsworth Center, Albany, New York, USA

## Abstract

From January 1, 1995, to December 31, 1997, we reviewed records of all New York City patients who had multidrug-resistant tuberculosis (MDRTB); we performed insertion sequence (IS) *6110*-based DNA genotyping on the isolates. Secondary genotyping was performed for low IS*6110* copy band strains. Patients with identical DNA pattern strains were considered clustered. From 1995 through 1997, MDRTB was diagnosed in 241 patients; 217 (90%) had no prior treatment history, and 166 (68.9%) were born in the United States or Puerto Rico. Compared with non-MDRTB patients, MDRTB patients were more likely to be born in the United States, have HIV infection, and work in health care. Genotyping results were available for 234 patients; 153 (65.4%) were clustered, 126 (82.3%) of them in eight clusters of >4 patients. Epidemiologic links were identified for 30 (12.8%) patients; most had been exposed to patients diagnosed before the study period. These strains were likely transmitted in the early 1990s when MDRTB outbreaks and tuberculosis transmission were widespread in New York.

Widespread transmission of multidrug-resistant Mycobacterium tuberculosis (MDRTB) strains occurred during the epidemic of the 1980s and early 1990s in New York City. Outbreaks were identified in many New York City hospitals and subsequently in New York State correctional facilities. Many of these outbreaks were associated with one strain (known as the "W" strain of TB) that was resistant to isoniazid, rifampin, ethambutol, and streptomycin and usually to kanamycin (1-5). However, other multidrug-resistant (MDR) strains were associated with outbreaks and nosocomial transmission during these years (6-8). Previous molecular epidemiology surveys in New York City showed that MDRTB was associated with clustered M. tuberculosis strains, which suggests recent transmission of the organism (9-11). The incidence of tuberculosis (TB) and MDRTB has been decreasing rapidly in New York City since 1992, when an enhanced Tuberculosis Control Program was implemented. The number of TB cases decreased 21.5% by 1994 (from 3,811 in 1992 to 2,995 in 1994), and MDRTB cases decreased 60% (from 441 to 176) (12,13). Since 1994, no outbreaks of MDRTB have been documented in the city.

To better understand the epidemiology of MDRTB, the New York City Tuberculosis Control Program began DNA genotyping of MDRTB strains from new cases in 1995. The objectives were to provide descriptive molecular epidemiology of MDRTB cases in the city during 1995-1997 and to identify predominant MDR strains present during these years, as well as the extent and risk factors for clustering among these cases.

## Methods

### Patient Selection

All patients with MDRTB (M. tuberculosis isolate resistant to at least isoniazid and rifampin) confirmed as TB cases from January 1, 1995, to December 31, 1997, in New York City were included. Demographic and clinical data were obtained from the New York City Tuberculosis Case Registry. The Registry's data were obtained from patient interviews and medical record reviews at the treatment or residential facilities by trained case managers using standardized data collection instruments and from contact investigations for each pulmonary case.

Susceptibility results were reviewed for the following TB treatment drugs: isoniazid, rifampin, pyrazinamide, ethambutol, streptomycin, and rifabutin (first-line drugs) and fluoroquinolone (usually ciprofloxacin or oflaxacin), kanamycin or amikacin, capreomycin, ethionamide, para-aminosalicylic acid, and cycloserine (second-line drugs). Susceptibility tests were done by Bactec radiometric method (Becton Dickinson and Co., Sparks, NY) for first-line drugs, except rifabutin (14), for most isolates and with standard proportion method with Middlebrook 7H10 media for both first- and second-line drugs for all isolates (15). Most of these tests were conducted at two reference laboratories, the New York City Department of Health and the New York State Department of Health, Wadsworth Center.

As part of routine surveillance, we reviewed the clinical histories of all pulmonary TB patients who had a negative acid-fast bacilli smear and only one positive M. tuberculosis isolate from a respiratory source. This review was to determine the accuracy of the culture result and to rule out laboratory error. If laboratory error was suspected for other types of specimens, clinical and laboratory data for patients were reviewed. Laboratory error was defined as a false-positive M. tuberculosis culture result that was caused by specimen mislabeling or laboratory cross-contamination, as evaluated by a described method (10). These patients were not counted as having verified cases of TB and were excluded from the analysis.

### Definitions

Patients were defined as having had prior treatment for TB if 1) drug-susceptible M. tuberculosis isolates were identified before the drug-resistant isolates that qualified the patients for this study; 2) they had documentation of previous TB disease or treatment; or 3) they had received >30 days of treatment with anti-TB drugs before collection of the specimen that grew MDR M. tuberculosis.

Patients were considered HIV seropositive when a positive HIV antibody test result was documented in the medical record or when AIDS was diagnosed before the TB diagnosis. The MDRTB diagnosis date was defined as the collection date of the first specimen from which an MDR M. tuberculosis isolate was cultured. Homelessness was defined as being in a public or private shelter or having no address at the time of the MDRTB diagnosis. Information about injection drug use within the 12 months before diagnosis was elicited from direct patient interviews and medical record reviews.

### Epidemiologic Investigations

Trained case managers obtained information about suspected and confirmed nosocomial and community exposure from patient interviews, contact investigations, and medical record reviews at the treatment or residential facilities. Probable nosocomial transmission was considered if the newly infected patient was in the same section of an institution as another patient who had an identical M. tuberculosis strain and was infectious (i.e., the patient had a positive culture from a respiratory site) at least 30 days before disease onset in the newly infected patient.

Community transmission was considered probable if either of the following occurred: 1) A patient was exposed to another patient who had the identical M. tuberculosis strain and was infectious (i.e., had a positive culture from a respiratory site) at least 30 days before disease onset in the subsequent patient. The exposure would have occurred in a home, single-room occupancy hotel, homeless shelter, or another noninstitutional setting. 2) The patient named another patient as a contact whose M. tuberculosis isolate had the same DNA pattern or who had MDRTB, but DNA genotyping result was not available.

Transmission could have been from a patient whose condition was diagnosed before the study period. If evidence of nosocomial or community transmission was found, patients had an epidemiologic link. The source patient was not considered to have an epidemiologic link.

During 1995 through 1996, nosocomial transmission was suspected at a hospital where the same MDR strain (i.e., identical insertion sequence [IS] 6110 band patterns) was found in six patients. Hospital floor, ward, and bed information and computerized outpatient clinic records from 1990 to 1996 were analyzed for temporal and spatial overlap among these patients. Medical records were reviewed for patient breaches of isolation protocol during hospitalization. Additional social and demographic information was collected through questionnaires. Specifically, patients were asked with whom and where they spent considerable time, and names of additional social contacts were requested. Patients were asked where and how they thought they had been exposed to TB.

### IS6110 DNA Genotyping and Other Molecular Studies

From 1995 through 1997, one M. tuberculosis isolate from each patient with MDRTB in New York City was sent to the Public Health Research Institute Tuberculosis Center, where DNA fingerprint analysis, based on IS6110 Southern blot hybridization pattern, was performed by using a standardized protocol (16). The Southern hybridization patterns were compared on a Sun Sparc5 Workstation (Sun MicroSystems, Santa Clara, CA), using Bio Image Whole Band Analyzer software version 3.4 (Bio Image, Ann Arbor, MI). Previously described methods were used to classify isolates (17). IS6110 banding patterns, which were similar to a parent strain but differed by one or two hybridization bands, were denoted by the addition of a number to the cluster letter (e.g., W, W1, P, or P1).

Secondary genotyping was performed by using spacer oligonucleotide typing (spoligotyping) and DNA sequencing of target gene regions that confer drug resistance. Spoligotyping and DNA sequencing of target gene regions used previously described methods (18-21).

If M. tuberculosis isolates had identical IS6110 band patterns, they were considered clustered. However, identical IS6110 patterns with less than six bands were not considered clustered, unless secondary DNA analysis confirmed a match, as noted in the results.

### Data Analysis

To examine how MDR patients differed from non-MDR patients, study subjects were compared to persons who had culture-positive TB diagnosed during the same period but were not included in this study. Descriptive analysis was performed for all study patients according to drug resistance patterns, DNA patterns, prior TB treatment, social and demographic variables, and evidence of nosocomial and community transmission. The Wilcoxon rank-sum test was used to compare medians of continuous variables, and the Pearson chi-square test was used to compare categorical data. Unconditional logistic regression was used to assess crude odds ratios and 95% confidence intervals for the association between potential risk factors and clustering. Statistical Analysis System Software (Release 8.01, SAS Institute, Inc., Cary, NC) was used for all data analyses. Statistical significance was set at a two-sided 5% level.

## Results

From 1995 through 1997, a total of 6,228 cases of TB were confirmed in New York City. Cultures from 5,136 (82.4%) persons were positive for M. tuberculosis. Of these, susceptibility results were available for 4,955 (96.5%); 241 (4.9%) persons had MDRTB. Findings of MDR for 11 additional isolates resulted from laboratory error (10 sputum and 1 urogenital); they were excluded from further analyses. The 241 patients made up 4.4% (106 of 2,445), 3.9% (81 of 2,053), and 3.1% (54 of 1,730) of all verified patients who had TB from 1995, 1996, and 1997, respectively. Table 1 presents a comparison of the demographic characteristics of these patients to those of culture-positive non-MDRTB patients from the same time period in New York City for whom drug susceptibility results were available. Compared with patients with culture-positive non-MDRTB during the same period, MDR patients were more likely to be born in the United States, have HIV infection, and be health-care workers, homeless, and injection drug users. MDR patients were more likely to have respiratory specimens positive for acid-fast bacilli and were less likely to be Asian. By further stratification, none of Asian MDRTB patients were born in the United States, and 68.7% of U.S.-born MDRTB patients were HIV infected.

**Table 1:** Comparison of social and demographic characteristics of multidrug-resistant (MDR) tuberculosis patients and non-multidrug-resistant tuberculosis patients, New York City, 1995-1997

	MDR (n=241)		Non-MDR (n=4,714)		
Characteristic	No.	%	No.	%	p value
Yr of diagnosis					
1995	106	44.0	1,816	38.5	0.0898
1996	81	33.6	1,586	33.6	0.9912
1997	54	22.4	1,312	27.8	0.0660
Median age (range), yrs	41 (5–85)	41 (0–100)	0.3071		
Male sex	147	61.0	3,020	64.1	0.3333
U.S.-born	166	68.9	2,483	52.7	<0.001
HIV serostatus					
Positive	128	53.1	1,478	31.4	<0.001
Negative	75	31.2	1,759	37.3	0.0521
Unknown	38	15.8	1,477	31.3	<0.001
Race/ethnicity					
Asian	24	10.0	817	17.3	0.0029
Hispanic	75	31.1	1,266	26.9	0.1461
Black non-Hispanic	105	43.6	2,089	44.3	0.8200
White non-Hispanic	37	15.4	542	11.5	0.0692
Health-care worker	15	6.2	109	2.3	0.0001
Homeless	28	11.6	344	7.3	0.0130
Injection drug user	33	13.7	275	5.8	<0.001
Disease site					
Pulmonary only	176	73.0	3,397	72.1	0.7440
Extra-pulmonary only	35	14.5	894	19.0	0.0848
Pulmonary + extra-pulmonary	30	12.5	423	9.0	0.0679
Specimen AFB smear-positive^a^	141	68.5	2,129	55.7	0.0003
Cavitary lesion(s)^b^	47	22.8	740	19.4	0.2247

^a^Respiratory specimen during the 30 days after initial specimen for bacteriologic test was taken. Excludes those who had only extrapulmonary disease. ^b^Excludes those with extrapulmonary disease only.

Strains were resistant to a median number of 6 drugs (range 2-10). Eight (3.3%) patients had strains of M. tuberculosis that were resistant to isoniazid and rifampin only, and 146 (60.6%) had isolates that were also resistant to one or more second-line anti-TB drugs. Most of these strains were also resistant to rifabutin. Twenty-four (10%) patients had received prior treatment for TB. Compared with patients who had no prior treatment, patients who had received such treatment were significantly older (median age 46 years vs. 41 years, p=0.010) and had less drug resistance (median 5 drugs versus 6, p=0.042). Patients with prior treatment were less likely to be born in the United States (45.8% vs. 71.4%, p=0.001) and were less likely to be HIV infected (33.3% vs. 55.3%, p=0.041). Patients who had received prior treatment did not differ from those who had no prior treatment according to gender, race or ethnicity, occupation, and histories of alcohol or drug abuse and homelessness.

DNA Genotyping AnalysisOf 241 MDR patients, 234 (97%) had IS6110 fingerprint patterns. Ninety-two different patterns were identified (band range 2-22). Thirty-six (15.4%) of 234 isolates had patterns with five or fewer IS6110 bands. Five were in one cluster, the C strain, and all had the same spoligotype (700036777760731). Two were clustered as a four-band strain with the same spoligotypes, and three other strains had unique genotypes. Twenty-six strains had an identical two-band IS6110 pattern designated as H; 25 of the 26 were resistant to pyrazinamide. All 17 with available results had identical spoligotypes (777776777760601); 18 of the 20 strains that were tested had identical pncA genotype (Nt70; G deletion). One pyrazinamide-susceptible strain had the wild-type pncA genotype, and one resistant strain had a different pncA genotype (139; GTG>GCG, Val>Ala). On the basis of the results, 18 of the 26 H strains were considered clustered. Thus, 25 of the 36 isolates with low IS6110 copies were considered clustered.

**Figure 1:**
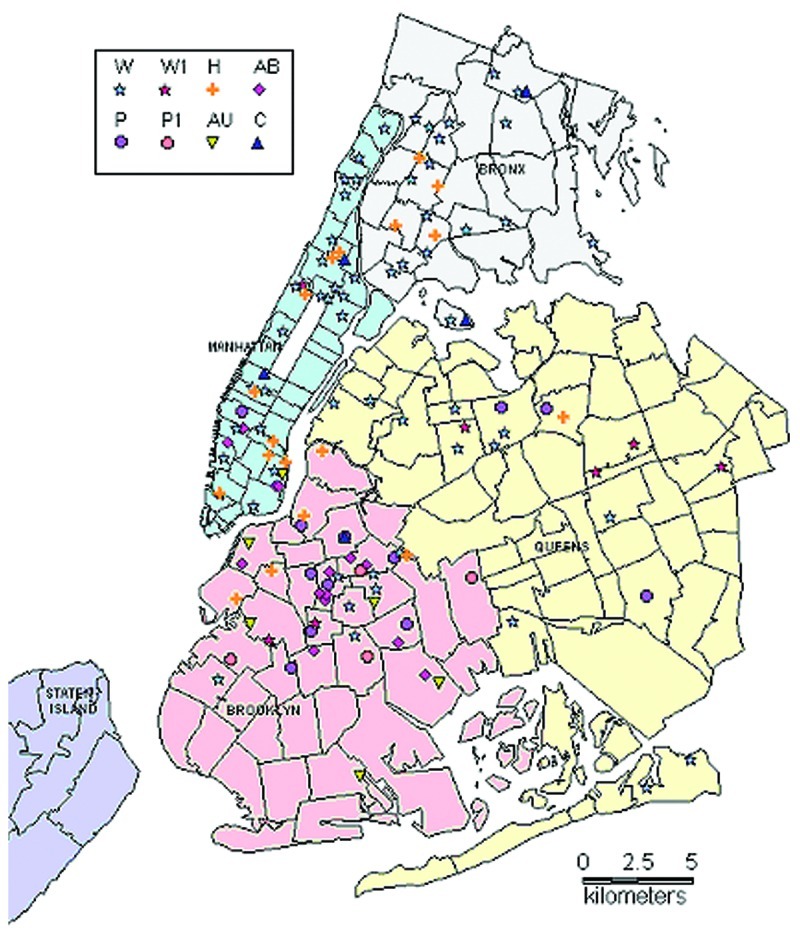
Geographic distribution of patients in major multidrug-resistant tuberculosis clusters, New York City, 1995-1997.

**Figure 2:**
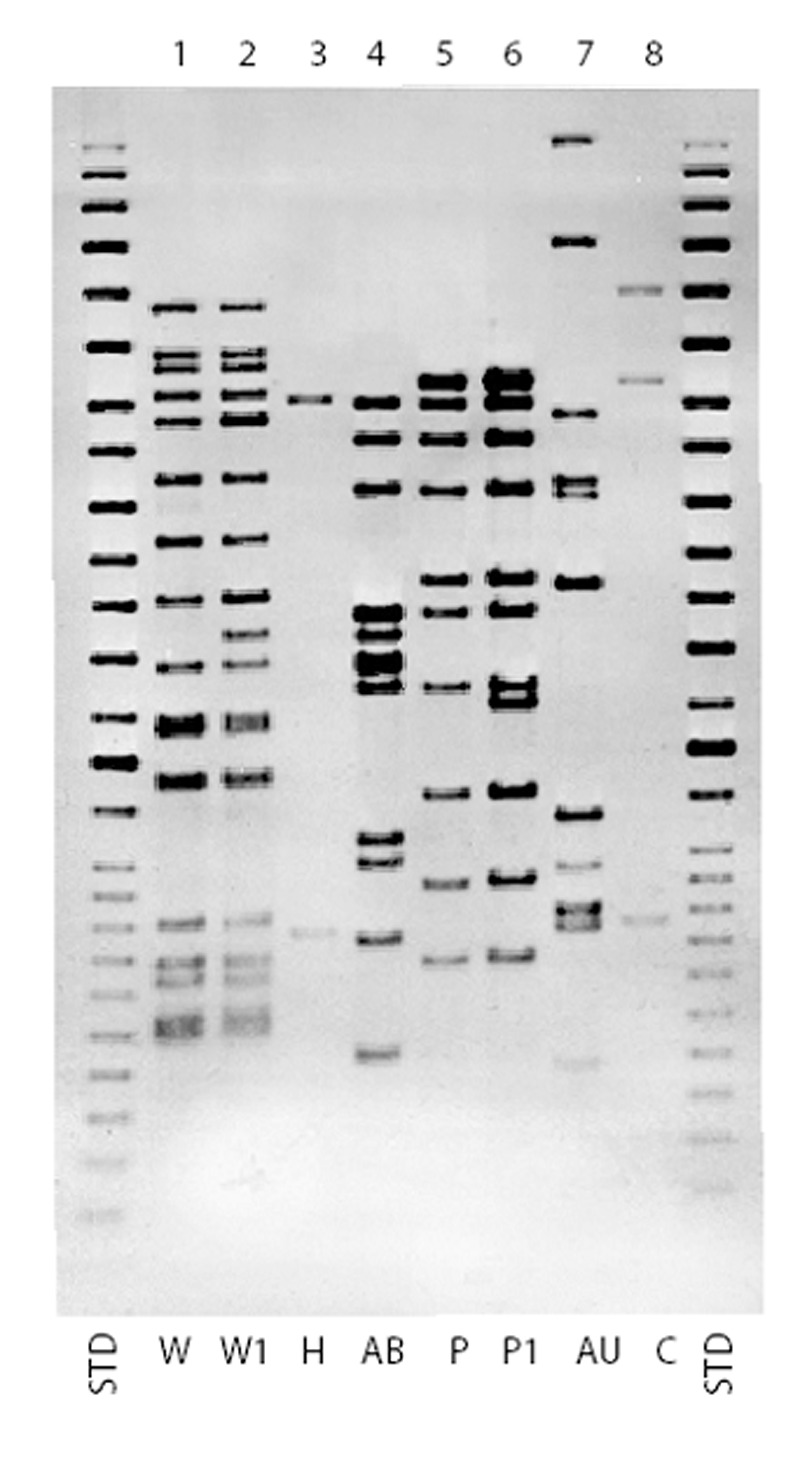
Insertion sequence (IS) 6110 Southern blot hybridization patterns for major multidrug-resistant Mycobacterium tuberculosis strains, New York City, 1995-1997. STD, standard.

Of 234 patients with DNA results, 153 (65.4%) were grouped into 19 clusters: 6 clusters with 2 cases each; 5 clusters with 3 cases each; and 8 different clusters with 4, 5, 6, 7, 13, 14, 18, and 59 cases each. The eight clusters had 126 (52.2%) of 241 MDRTB patients from the study period. Table 2 shows the distribution of these eight strains during 1995 through 1997 with social, demographic, and epidemiologic link information. Figure 1 shows the geographic distribution, and Figure 2 shows the IS6110 patterns of these eight strains.

**Table 2:** Social and demographic characteristics of patients in predominantly multidrug-resistant tuberculosis clusters, New York City, 1995–1997

	Clustered strain (n=234)									
Characteristics	W	W1	H	AB	P	AU	C	P1	Other	Unique RFLP^a^
No. of patients	59	7	18	14	13	6	5	4	27	81
No. of bands	18	19	2	11	11	10	3	11	4–18	2–22
Known epidemiologic links										
Nosocomial	6	1	0	0	0	0	0	0	0	0
Community	4	1	2	7	1	2	0	0	1	5
Age (median, in yrs)	41	41	37	42	43	40	37	37	41	42
Male	41	3	7	7	7	4	4	3	17	49
Race/ethnicity										
Asian	2	1	1	0	1	0	1	0	0	16
Hispanic	18	2	10	1	3	1	3	2	9	24
Black, non-Hispanic	25	1	6	12	9	1	1	2	15	31
White, non-Hispanic	14	3	1	1	0	4	0	0	3	10
U.S.-born	46	4	15	13	10	5	3	3	21	42
HIV positive	42	1	14	10	4	4	3	1	17	29
History of—										
Homelessness	6	0	1	8	3	0	2	0	2	5
Alcohol abuse	11	1	1	3	4	0	0	0	5	9
Injection drug use	9	0	2	6	0	1	0	1	5	8
Prior tuberculosis treatment	0	0	1	0	1	1	2	0	5	12
Health-care worker	4	3	1	0	1	0	1	0	2	2
Borough of residence										
Manhattan	23	1	8	4	1	1	3	0	10	19
Bronx	17	0	4	0	0	0	1	0	2	8
Brooklyn	7	2	5	10	9	5	1	4	11	30
Queens	12	4	1	0	3	0	0	0	4	23
Staten Island	0	0	0	0	0	0	0	0	0	1

^a^RFLP, restriction fragment length polymorphism.

Epidemiologic links were identified for 30 (12.8%) of the 234 patients with genotyping results; most had been exposed to patients diagnosed before the study period. Twenty-five (19.8%) of 153 patients clustered by DNA genotyping were epidemiologically linked; 18 (72%) had probable community transmission, and 7 (28%) had probable nosocomial transmission. All nosocomial links were to patients whose conditions were diagnosed before the study period. Seven community transmission links were to patients from the study period, and 11 were to patients whose diseases were diagnosed before the study period. Epidemiologic links of community transmission were identified for 5 (6.2%) of 81 nonclustered patients; all were links to persons whose conditions were diagnosed before the study period. Of the 23 community links, 3 were to household members, 4 to nonhousehold relatives, and 7 to friends. One was linked to another case in a single-room occupancy hotel; seven were linked in a crack den, and one had an unknown exposure site.

Table 3 shows a comparison of patients clustered by DNA analysis to those nonclustered according to various demographic and clinical characteristics. Factors significantly associated with clustering were HIV infection and birth in the United States. There was no difference in proportion clustered by year. Patients with histories of prior treatment and Asian patients were significantly less likely (odds ratio [OR] = 0.40, 95% confidence interval [CI] = 0.17 to 0.98 and OR=0.18, 95% CI-0.06 to 0.53, respectively) to be in a cluster. Patients in clusters were 3 times more likely to have epidemiologic links than those not in clusters. In a subanalysis that included only non-U.S.-born patients who had a known date of entry to the United States, clustering was significantly associated (OR=1.09, 95% CI=1.02, l.16; p=0.01) with longer time of residence in the United States.

**Table 3:** Risk factors associated with clustering of multidrug-resistant tuberculosis cases, New York City, 1995–1997 (n=234)

Characteristic	Clustered (n=153) No. (%)	Nonclustered (n-81) No. (%)	Crude OR		95% CI
Median age in yr (range)	41 (5–85)	42 (22–77)		0.99	0.98, 1.02
Male sex	93 (60.8)	49 (60.5)		1.01	0.58, 1.76
U.S.-born	120 (79.0)	42 (51.9)		3.48	1.94, 6.25
Median years of residence in United States^b^	12 (0–47)	6.5 (0–24)		1.09	1.02, 1.16
HIV serostatus					
Positive	96 (62.8)	29 (35.8)		2.81	1.52, 5.22
Negative	40 (26.1)	34 (42.0)		1.00	
Unknown	17 (11.1)	18 (22.2)		0.80	0.36, 1.80
Race/ethnicity					
Asian	6 (3.9)	16 (19.8)		0.18	0.06, 0.53
Hispanic	49 (32.0)	24 (29.6)		1.00	
Black non-Hispanic	72 (47.1)	31 (38.3)		1.14	0.60, 2.17
White non-Hispanic	26 (17.0)	10 (12.4)		1.27	0.53, 3.06
Health-care worker	12 (7.8)	2 (2.5)		3.36	0.73, 15.40
Homeless	22 (14.4)	5 (6.2)		2.55	0.92, 7.02
Injection drug use^c^	24 (15.7)	8 (9.9)		1.70	0.73, 3.97
Prior treatment history	10 (6.5)	12 (14.8)		0.40	0.17, 0.98
Having epidemiologic link^d^					
Nosocomial	7 (4.6)	0 (0)		2.97	1.02, 9.26
Community	18 (11.8)	5 (11.1)			
No link	128 (83.7)	76 (88.9)		1.00	
Year of diagnosis					
1995	69 (45.1)	32 (39.5)		1.00	
1996	54 (35.3)	27 (33.3)		0.93	0.47, 1.81
1997	30 (19.6)	22 (27.2)		0.63	0.30, 1.34

^a^OR, odds ratio; CI, confidence interval. ^b^Excludes non-U.S.-born patients.  ^c^Injection drug use within 12 months before diagnosis. ^d^Compared epidemiologic link with no epidemiologic link

### Epidemiology of Predominant MDR Strains

## Discussion

During the 3-year period, 241 (3.9%) of all 6,228 TB cases in New York City and 241 (4.9%) of all 4,995 M. tuberculosis culture-positive patients with susceptibility had MDR strains. MDRTB patients were more likely to have acid-fast bacilli visible on microscopic examination of respiratory specimens and thus were more infectious. MDRTB was more common in patients who were born in the United States, HIV infected, non-Asian, or health-care workers. The finding of greater prevalence of HIV infection in MDRTB patients compared with non-MDRTB patients is likely due to several reasons. The initial outbreaks during which these strains were transmitted mostly involved HIV-infected persons (1-8). A large number of HIV-infected patients were likely infected in those outbreaks. HIV-infected patients progress from infection to disease at a much higher rate than non-HIV-infected persons.

Most patients in this study had primary MDRTB caused by a few strains. The proportion of patients clustered in this cohort is much higher than in previously reported New York City patients. Few demographic and clinical characteristics were associated with clustering. According to previous citywide surveys of all patients who had cultures that were positive for TB, the proportions of clustered patients who had TB were 37% in 1991 and 32.4% in 1994 (10,11). Another investigation from one hospital in the city found similar results for patients during 1989-1991 (9). A more recent survey from 1997 in New York City found that, for persons born in the United States, the proportion clustered had not decreased (23). In all these surveys, MDRTB was associated with clustering in multivariate analyses. The higher proportion of MDRTB clustering seen in this study cannot be explained by the exclusion of low-band patients in previous citywide surveys. When we exclude low-band patients from our analysis, we still have a similar proportion of clustering (128 [64.6%] of 198 isolates with less than five bands). Our proportion of MDRTB clustering is also higher than that reported from other U.S. cities and other industrialized countries, where approximately 18% to 49% of clustering has been observed (24-28). However, few patients in these studies had MDRTB. The inclusion of MDRTB patients only in this study may have contributed to this difference. A study conducted during 1995 to 1997 by Moro et al. in Italy showed 74.2% clustering among MDRTB patients, compared with 39.3% among non-MDR cases (29). Our study reiterates that a few, highly resistant strains were transmitted widely in New York City during the late 1980s and early 1990s.

Strains W, W1, P, P1, and H were transmitted in the early 1990s during the period of MDRTB outbreaks in New York City because five of the eight major clustered strains were associated with hospital outbreaks during that time (1-8). Few patients in this cohort had epidemiologic links, but most of these links were to patients whose diseases were diagnosed before the study period. Most health-care workers (10 of 14 with DNA results) had one of the known outbreak strains, but only 2 could be linked to facilities where nosocomial transmission occurred.

In addition to the nosocomially transmitted strains, we identified a large cluster that may have been transmitted in a community of persons who were HIV infected, homeless, and drug users. Before this study period, at least 14 additional MDRTB patients with this strain had been identified and confirmed by genotyping from 1989 through 1994. Six of these patients were from the same borough, and four were from the same health district as many of the patients in 1995-1997. This strain was transmitted over many years among drug users who were frequenting crack dens in the same neighborhood. Since many of these venues were closed in the late 1990s, this social group was disrupted, and transmission was interrupted. The AB strain has been found in only two new patients during 1998-2001, one in 1998 and the other in 2001. The patient from 2001 had epidemiologic links to a patient from 1995.

Five patients had the C strain, which has three IS6110 copies. This M. tuberculosis strain is the most common in the city. Most of the C strains in the city share the same spoligotype and pTBN12-based RFLP pattern and are clonal (30, New York City Department of Health and Public Health Research Institute, unpub. data). Most of the C strains have been drug-susceptible; however, we identified C strains with varying drug-resistant patterns, occasionally in clusters (30, New York City Department of Health and Public Health Research Institute, unpub. data). The MDR strains in this period appear to be a recent cluster, or each may have acquired drug resistance separately.

MDRTB continues to decline in New York City at a rapid rate, with only 38, 31, and 25 new cases identified in 1998, 1999, and 2000, respectively (31). However, most of the major strains found in this investigation continued to be identified in new MDRTB patients in New York City from 1998 through 2001 (New York City Department of Health and Public Health Research Institute, unpub. data). Most nonclustered patients had primary drug-resistant TB. The improved Tuberculosis Control Program, which was implemented in 1992 with aggressive case management and direct observation of anti-TB therapy for most patients, quickly curtailed the development of newly acquired drug resistance. Since primary and acquired drug resistance and MDRTB, in particular, were prevalent before 1995 (32,33), many MDRTB strains likely were disseminated in the community because most patients in this cohort with unique strains had no histories of prior treatment.

In this study, we may have underestimated the number of cases that had nosocomial and community epidemiologic links. We did not use medical record reviews of hospitalizations before the diagnosis of MDRTB for all the patients to identify potential nosocomial exposures. Many patients died before identification of MDRTB; therefore, interviews could not be conducted to identify potential nosocomial and community exposures before diagnosis of TB. The outbreaks associated with the W and W1 strains were well investigated and publicized, and staff were aware of the locations of the outbreak hospitals. This fact may have allowed for easier identification of epidemiologic links in these patients. In the AB community outbreak cluster, most epidemiologic links were identified from the detailed interviews with the few patients who were still alive. Traditional contact investigations did not identify these links in this subpopulation. This observation underscores that other methods, such as ongoing surveillance for unusual patterns of disease and unusual patient characteristics, should also be used to identify possible transmission in the community. Prospective DNA typing of all isolates can also supplement traditional contact investigation methods.

The molecular analysis of the MDRTB strains in New York City during these years demonstrated that the improved Tuberculosis Control Program has reduced dramatically the transmission of these strains. These investigations have also established important baseline data for the study of the epidemiology of MDRTB over the next decades.
